# Xylonolactonase from *Caulobacter crescentus* Is a Mononuclear Nonheme Iron Hydrolase

**DOI:** 10.1021/acs.biochem.1c00249

**Published:** 2021-10-11

**Authors:** Johan Pääkkönen, Leena Penttinen, Martina Andberg, Anu Koivula, Nina Hakulinen, Juha Rouvinen, Janne Jänis

**Affiliations:** † Department of Chemistry, University of Eastern Finland, P.O. Box 111, FI-80101 Joensuu, Finland; ‡ VTT Technical Research Centre of Finland Ltd, P.O. Box 1000, FI-02044 VTT Espoo, Finland

## Abstract

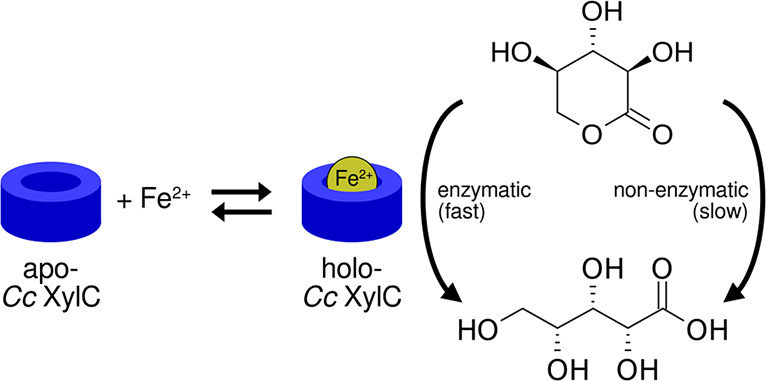

*Caulobacter crescentus* xylonolactonase (*Cc* XylC, EC 3.1.1.68) catalyzes an intramolecular ester bond hydrolysis over a nonenzymatic acid/base catalysis. *Cc* XylC is a member of the SMP30 protein family, whose members have previously been reported to be active in the presence of bivalent metal ions, such as Ca^2+^, Zn^2+^, and Mg^2+^. By native mass spectrometry, we studied the binding of several bivalent metal ions to *Cc* XylC and observed that it binds only one of them, namely, the Fe^2+^ cation, specifically and with a high affinity (*K*_d_ = 0.5 μM), pointing out that *Cc* XylC is a mononuclear iron protein. We propose that bivalent metal cations also promote the reaction nonenzymatically by stabilizing a short-lived bicyclic intermediate on the lactone isomerization reaction. An analysis of the reaction kinetics showed that *Cc* XylC complexed with Fe^2+^ can speed up the hydrolysis of d-xylono-1,4-lactone by 100-fold and that of d-glucono-1,5-lactone by 10-fold as compared to the nonenzymatic reaction. To our knowledge, this is the first discovery of a nonheme mononuclear iron-binding enzyme that catalyzes an ester bond hydrolysis reaction.

Metal cations are essential for the catalytic activity of several enzymes; thus, their accurate identification, binding affinity determination, and coordination characteristics are essential in the understanding of enzyme function.^1^ High-resolution native mass spectrometry (MS) is a powerful method to characterize metal ion binding to folded proteins with a high accuracy.^2^ When we used native MS to characterize the xylonolactonase from *Caulobacter crescentus* (*Cc* XylC), we observed unexpectedly that it binds only the Fe^2+^ cation with a high affinity and specificity, suggesting that the previous understanding of the metal ion binding to this enzyme is inadequate.

*Cc* XylC uses d-xylonolactone as a substrate and produces d-xylonic acid.^3^d-Xylonolactone exists as two isomers, 1,4- and 1,5-lactones, which can interconvert via a short-lived bicyclic intermediate.^4,5^ Therefore, it is difficult to estimate whether 1,4- or 1,5-lactone would be a preferable substrate for *Cc* XylC. However, the crystal structures of the homologous SMP30 protein have shown well-ordered electron densities for the six-membered ring ligands, suggesting that the binding of 1,5-lactone would be preferable also for *Cc* XylC.^6^ Also, Jermyn has found that 1,5-lactone is a true substrate for the homologous gluconolactonase from *Pseudomonas fluorescens*.^4^

This lactonase-catalyzed reaction is the second step in the oxidative, nonphosphorylative d-xylose (Dahms or Weimberg) pathway in bacteria. The Dahms pathway can also be utilized for the production of several platform chemicals such as ethylene glycol, glycolic acid, lactic acid, and 1,4-butanediol starting from xylose-rich biomass fractions.^3^ On the basis of the amino acid sequence homology, *Cc* XylC is a member of the senescence marker protein 30 (SMP30) protein family, which includes several aldonolactonases. The amino acid sequence search within the Protein Data Bank (PDB) results in a few enzyme structures, which possess homologous sequences. These include, for example, SMP30 gluconolactonases (31–34% identity), luciferin-regenerating enzyme (33% identity), *Xanthomonas campestris* gluconolactonase (30% identity), and *Linaria vulgaris* diisopropylfluorophosphatase (26% identity). All solved SMP30 family members share an overall tertiary structure consisting of a six-blade β-propeller with a central channel, where a bivalent metal ion is tri- or tetracoordinated by asparagine, aspartate, or glutamate side chains. Ca^2+^, Zn^2+^, and Mg^2+^ metal ions have been used in the crystal structure refinements.^6−10^ Thus, the previous studies suggest that this enzyme family is capable of binding different bivalent metal cations. To obtain further information about the metal ion binding of *Cc* XylC, we chose to use native MS, since it allows the metal binding stoichiometry, affinity, and specificity to be directly observed.

The mass spectra of *Cc* XylC were measured by using a high-resolution Fourier transform ion cyclotron resonance (FT-ICR) instrument (Bruker Solarix XR), equipped with an electrospray ionization (ESI) source. The elemental formula obtained from the amino acid sequence of *Cc* XylC (omitting the initial methionine residue) is C_1422_H_2158_N_378_O_422_S_7_, and the corresponding theoretical most abundant isotopic mass is 31 524.76 Da. The mass spectrum of the denatured *Cc* XylC (Figure S1) gave the most abundant mass of 31 524.89 ± 0.10 Da (mean ± standard deviation), averaged over the observed charge state distribution, consistent with the theoretical value. In the denatured state, no metal ion binding to *Cc* XylC was observed, as expected. In contrast, when *Cc* XylC was measured in the native state, an additional signal was surprisingly observed at 31 577.49 Da in the deconvoluted mass spectrum. The mass difference of ∼53 Da corresponds to the binding of a single Fe^3+^ ion (theoretical mass 31 577.67 Da, assuming a formal removal of three hydrogens upon iron ion binding). This was an unexpected observation, since no iron binding has been suggested for the other SMP30 family lactonases in any previous studies. To obtain a spectrum of the apoprotein, the protein sample was treated with ethylenediaminetetraacetic acid (EDTA) (at least a 20-fold molar excess) to chelate any metal ions before sample desalting. Following the EDTA treatment, only the 31 524.56 Da signal remained in the spectrum (Figure 1A).

**Figure 1:**
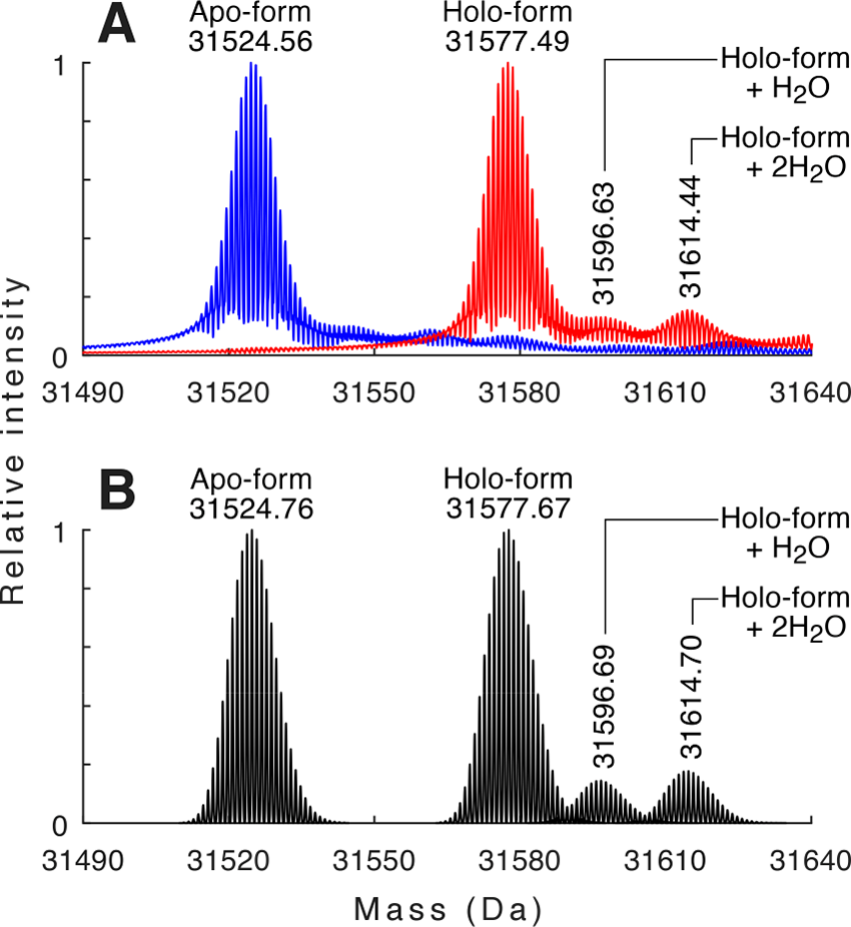
(A) Deconvoluted native mass spectra of *Cc* XylC in apo-form (blue) and holo-form (red). (B) Calculated mass spectra of the corresponding forms of *Cc* XylC. The most abundant isotopic masses are indicated.

To study whether the iron binding to *Cc* XylC was specific, the binding of six other metals was also tested. The initial metal binding tests were done by adding either Mg^2+^, Ca^2+^, Fe^2+^, Fe^3+^, Co^2+^, Ni^2+^, Cu^2+^, or Zn^2+^ at 10 μM to apo-*Cc* XylC (EDTA-treated) at 1 μM. All metal ions were added as their analytical grade chloride salts, dissolved in 10 mM ammonium acetate solution. Native MS measurements indicated that only Fe^2+^ was bound strongly and specifically to *Cc* XylC, while the other metals did not show even weak binding, except Cu^2+^, for which the native MS showed that up to four Cu^2+^ ions were able to bind to the protein. The Cu^2+^ binding most likely occurs through the four free cysteine residues and not to the active site of the enzyme.

Also, two minor signals corresponding to the additional binding of one and two water molecules to the iron-complexed holo-form of *Cc* XylC were observed (Figure 1A). These waters are likely coordinated to the metal ion and remain bound during the ionization process. Such water molecules are rarely detected in the gas phase (except in some small metal coordination complexes), due to their low enthalpy of dehydration, but have been previously detected, for example, for an *Escherichia coli* deaminase transition-state analogue complex.^11^ As the bound iron ion was observed as Fe^3+^, even though Fe^2+^ was originally added to the solution, we also tested if Fe^3+^ was capable of binding to the protein. Since no binding was observed with Fe^3+^, it is clear that iron binds to *Cc* XylC only in the oxidation state +2 but undergoes a rapid oxidation to +3 in the electrospray process. A similar behavior has been observed earlier with the heme-binding proteins.^12^ It was also observed that the iron oxidation during the electrospray process does not occur when water molecules are coordinated to the Fe^2+^ cation (Figure 1A).

To determine the iron binding affinity of *Cc* XylC, 1 μM apoenzyme was titrated with Fe^2+^ (up to 16 μM), and the fractional saturation was monitored. The fitting of the data to the specific, one-site binding model yielded a *K*_d_ value of (5.0 ± 1.3) × 10^–7^ M and *B*_max_ of 0.966 ± 0.009 with a 95% level of confidence (Figure 2).

**Figure 2:**
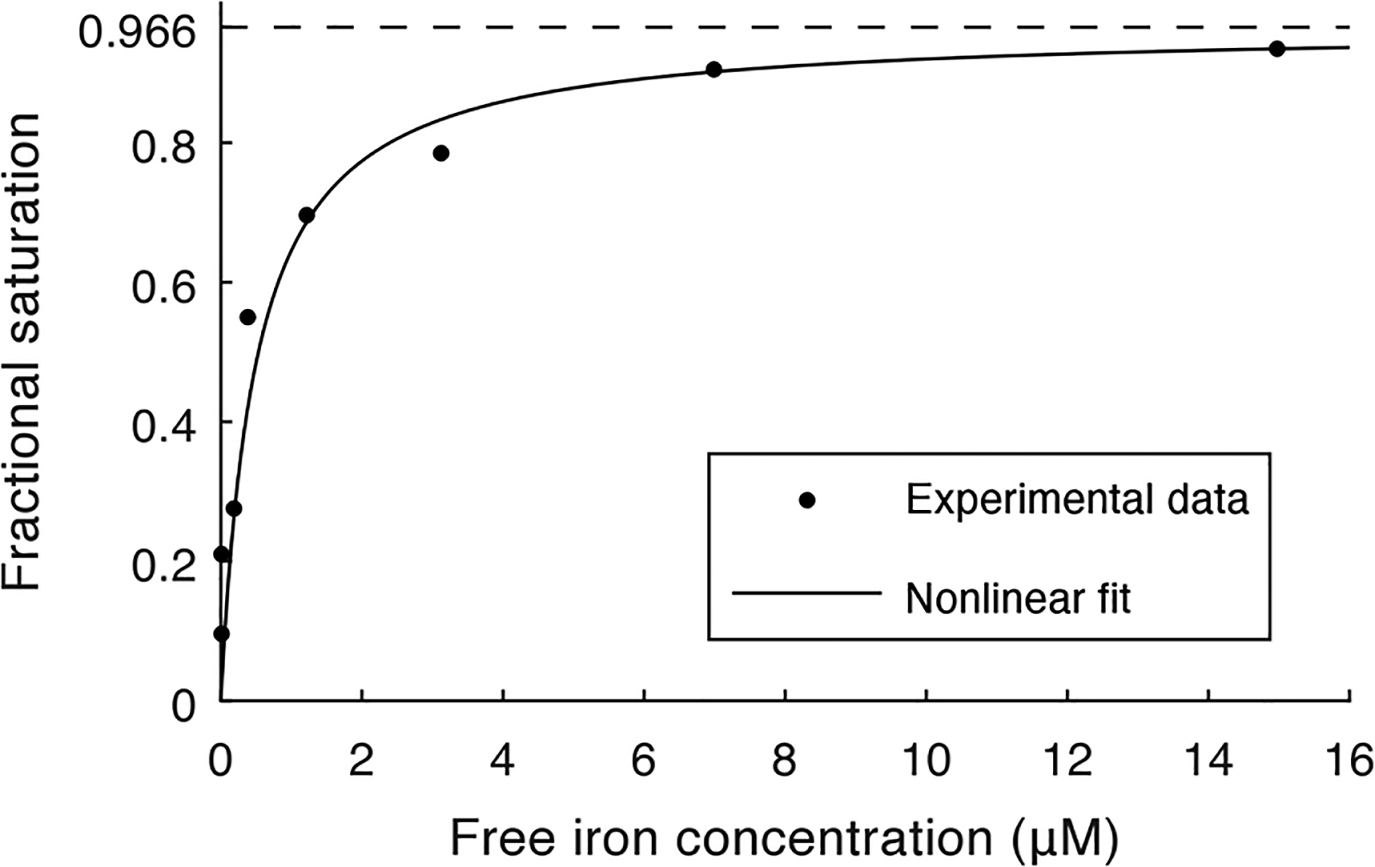
Titration of *Cc* XylC with Fe^**2**+^.

The influence of Fe^2+^ for enzymatic activity was also tested by following the lactone hydrolysis reaction (at the initial pH 6.9) with an ion trap mass spectrometer. Probably due to difficulties in the isolation of lactone isomers, only d-xylono-1,4-lactone and d-glucono-1,5-lactone were commercially available and used as substrates at the initial 0.25 mM concentration. Without an enzyme or metal present (nonenzymatic reaction), both xylono- and gluconolactones were hydrolyzed with the half-lives of 330 ± 40 and 45 ± 5 min (confidence intervals with a 95% level of confidence), respectively. The results are in agreement with the results of Jermyn,^4^ who found that the nonenzymatic hydrolysis of 1,5-lactone is faster as compared to 1,4-lactone and suggested that 1,4-lactone is isomerized via a bicyclic intermediate to 1,5-lactone, which is then hydrolyzed. This isomerization reaction is reversible and fast.^4,5^

The addition of 10 μM Fe^2+^ in the absence of enzyme slightly accelerated the hydrolysis of both substrates, suggesting that the bare metal ions can also promote the nonenzymatic hydrolysis reactions. Because the acceleration was greater for 1,4-lactone, this would suggest that metal ion could catalyze the isomerization reaction between 1,4-lactone and 1,5-lactone (Figure 3).

**Figure 3:**
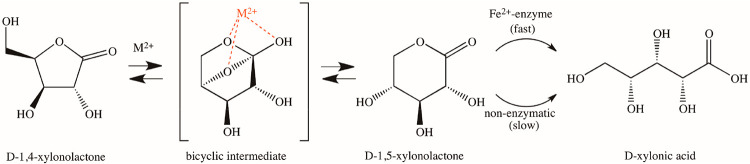
Suggested total reaction of d-xylonolactone hydrolysis.

We then performed the hydrolysis reactions in the presence of 10 μM Fe^2+^ and 0.5 μM *Cc* XylC, which resulted in a considerable increase in the hydrolysis rates for both lactones. The half-lives of xylono- and gluconolactones were reduced to 4.3 ± 0.3 and 4.8 ± 0.4 min, respectively, corresponding to the estimated *k*_cat_/*K*_M_ values of (5.4 ± 0.9) × 10^3^ and (4.9 ± 0.9) × 10^3^ M^–1^ s^–1^. Since the values are of the same magnitude, it can be concluded that the enzyme catalyzes the hydrolysis of both lactones equally and that the enzyme is not specific to either lactone (xylono- or gluconolactone). The reaction rate over the nonenzymatic hydrolysis was larger for d-xylono-1,4-lactone (100-fold) than for d-glucono-1,5-lactone (10-fold). The apparent reaction rate constants (pseudo-first-order) and the reaction half-lives are summarized in Table 1.

**Table 1:** Reaction Rate Constants and Half-Lives for *Cc* XylC-Catalyzed Hydrolysis of Xylono- and Gluconolactones

substrate (main isomer)	*Cc*XylC (μM)	Fe^2+^ (μM)	rate constant^a^^,^^b^*k* (s^–1^)	half-life^b^*t*_1/2_ (min)
d-xylono-1,4-lactone	0	0	(3.5 ± 0.5) × 10^–5^	330 ± 40
d-xylono-1,4-lactone	0	10	(8.3 ± 0.5) × 10^–5^	138 ± 8
d-xylono-1,4-lactone	0.5	10	(2.7 ± 0.2) × 10^–3^	4.3 ± 0.3
d-glucono-1,5-lactone	0	0	(2.6 ± 0.3) × 10^–4^	45 ± 5
d-glucono-1,5-lactone	0	10	(4.0 ± 0.6) × 10^–4^	29 ± 4
d-glucono-1,5-lactone	0.5	10	(2.4 ± 0.2) × 10^–3^	4.8 ± 0.4

a Pseudo-first-order.

b Confidence intervals with a 95% level of confidence.

Boer et al. have observed earlier^3^ that Ca^2+^ and Zn^2+^ increase the rate of d-xylonolactone hydrolysis. Because we did not observe the binding of these metal cations to *Cc* XylC, they must also speed up the reaction nonenzymatically by binding to the above-mentioned bicyclic reaction intermediate. This interaction is less specific for different bivalent metal ions. If *Cc* XylC utilizes preferably 1,5-lactone as the substrate, the presence of metal ions hastens the formation of 1,5-lactone and consequently also the enzymatic hydrolysis (Figure 3).

Studying the enzyme-catalyzed hydrolysis of an intramolecular ester bond in lactones is challenging because lactones are also nonenzymatically hydrolyzed to sugar acids by utilizing acid or base catalysis in a water medium. There is also evidence in the earlier studies that metal ions, especially bivalent metal ions, are able to weakly catalyze the ester bond hydrolysis.^13,14^ Therefore, careful experimental analyses are needed to dissect the actual roles of metal ions in catalysis. In this respect, the use of native mass spectrometry in analyzing the metal ion binding to *Cc* XylC has proven to be very essential. The results show that the enzyme is highly specific for Fe^2+^, and the affinity to other bivalent metal cations is very low. This result suggests that *Cc* XylC is a mononuclear nonheme iron enzyme. To the best of our knowledge, the other known examples of mononuclear iron enzymes are all oxidases, typically utilizing molecular dioxygen.^15,16^ In consequence, this study suggests that Fe^2+^ may exist as a catalytic metal also in hydrolytic enzymes. Further studies are needed to clarify if Fe^2+^ would be a catalytic metal ion also among other members of the SMP30 protein family.

## Supporting Information Available

Experimental procedures, additional mass spectra, iron affinity determination, hydrolysis reaction progress curves (PDF)

## Accession Codes

*Caulobacter crescentus* xylonolactonase C; UniProtKB accession code: A0A0H3C6P8 (A0A0H3C6P8_CAUVN)

## Abbreviations

*Cc* XylC: *Caulobacter crescentus* xylonolactonase C
ESI: electrospray ionization
FT-ICR: Fourier transform ion cyclotron resonance
SMP30: senescence marker protein 30.
