# Supplementary material for: Xylonolactonase from Caulobacter crescentus Is a Mononuclear Nonheme Iron Hydrolase
Source: Biochemistry. 2021 Oct 11;60(41):3046–9. doi: 10.1021/acs.biochem.1c00249 (PMC8529709; doi:10.1021/acs.biochem.1c00249)
Supplement: Supplementary file 1 — bi1c00249_si_001.pdf [file bi1c00249_si_001.pdf]

Supporting information

Xylonolactonase from *Caulobacter crescentus* Is a Mononuclear Nonheme Iron Hydrolase

Johan Pääkkönen*, Leena Penttinen*, Martina Andberg†, Anu Koivula†, Nina Hakulinen*, Juha Rouvinen*, and Janne Jänis*‡

* Department of Chemistry, University of Eastern Finland, P.O. Box 111, 80101 Joensuu, Finland

† VTT Technical Research Centre of Finland Ltd, P.O. Box 1000, 02044 VTT, Espoo, Finland

‡ Corresponding author – orcid.org/0000-0002-8446-4704, e-mail: janne.janis@uef.fi

Table of Contents

1 ESI FT-ICR MS experimental procedures . . . S2
2 ESI QIT MS experimental procedures . . . S8
3 Determination of iron affinity to *Cc* XylC . . . S9
4 Monitoring the nonenzymatic and enzymatic lactone hydrolysis . . . S10
5 References . . . S13

List of Tables

S1 ESI FT-ICR MS measurement parameters . . . S3
S2 ESI QIT MS measurement parameters . . . S8

List of Figures

S1 Mass spectrum of *Cc* XylC in denatured state . . . S4
S2 Charge state deconvolution of the denatured mass spectrum . . . S4
S3 Native mass spectrum of the untreated *Cc* XylC . . . S5
S4 Native mass spectrum of *Cc* XylC in apo-form . . . S5
S5 Native mass spectrum of *Cc* XylC in holo-form . . . S6
S6 Native mass spectrum of *Cc* XylC with Zn^{2+} present . . . S6
S7 Native mass spectrum of *Cc* XylC with Cu^{2+} present . . . S7
S8 Measured reaction progresses and fitted progress curves . . . S12

1 ESI FT-ICR MS experimental procedures

The instrument used for measuring protein mass spectra was a Bruker Solarix XR Fourier transform ion cyclotron resonance (FT-ICR) mass spectrometer (Bruker Daltonik GmbH, Bremen, Germany), equipped with an electrospray ionization (ESI) source. The ion source pressure was $\sim 5 \cdot 10^{-6}$ mbar, and the cell pressure was $\sim 3 \cdot 10^{-10}$ mbar. Important measurement parameters are shown in Table S1. Calibration was done using ES Tuning Mix (Agilent Technologies, Santa Clara, CA, USA).

The *Cc* XylC was expressed in *E. coli* and purified essentially as described by Boer et al.[1] For mass spectrometry measurements, the *Cc* XylC sample was buffer-exchanged to 10 mM ammonium acetate (ultra-pure in HPLC quality water, pH 6.9) using a PD-10 desalting column (GE Healthcare, Freiburg, Germany). The concentration of this solution was determined using UV absorbance at 280 nm, and it was diluted to approximately 1 µM.

Denaturing conditions were created by mixing enzyme solution, acetonitrile and acetic acid (49.5/49.5/1.0, *v*/*v*). A denatured mass spectrum (Figure S1) was obtained by measuring this solution. Charge state deconvolution (Figure S2) was done in GNU Octave[2] with a custom function which summed all the observed charge states after transforming them to neutral mass space and aligning the peaks. The neutral mass of the enzyme was calculated by averaging the observed masses of the isotopic peaks in each observed charge state (from 18+ to 33+) corresponding to the most abundant isotopic peak in the deconvoluted spectrum; the mean mass was 31,524.89 Da, and the standard deviation was 0.10 Da.

The native mass spectra were measured first with the enzyme solution in 10 mM ammonium acetate. This resulted in a spectrum (Figure S3) containing the previously observed apo-enzyme as well as another significantly strong signal, the mass of which corresponded to the binding of a single Fe^{3+} ion to the enzyme. The pure apo-enzyme was made by adding approximately 20 molar equivalents of EDTA to the enzyme sample before the buffer exchange, which yielded a spectrum (Figure S4) with no detectable signal of the holo-enzyme. Metal ions were introduced by adding the corresponding metal chloride solutions to the buffer-exchanged enzyme solution so that the concentration of the metal was 10 µM. With Fe^{2+} ions present, only the iron-complexed holo-enzyme was observed (Figure S5), whereas with Mg^{2+}, Ca^{2+}, Fe^{3+}, Co^{2+}, Ni^{2+} or Zn^{2+}, no significant change to the previous spectrum of the apo-enzyme was observed (Figure S6). With Cu^{2+} ions present, weak signals corresponding to one to four bound Cu^{2+} ions appeared (Figure S7); this was most likely due to the copper binding to free cysteines on the surface of the enzyme.

To determine the iron affinity to the *Cc* XylC, a series of solutions with differing Fe^{2+} concentrations was prepared. The enzyme concentration was kept constant, at approximately 1 µM, and Fe^{2+} concentration was varied from 7.8 nM to 16 µM, increasing in geometric progression. Native mass spectra were measured as before. At below 125 nM Fe^{2+}, the

holo-enzyme could not be observed, and so those spectra were discarded. At 16 μM Fe^{2+}, there was a small amount of apo-enzyme left even though previously it disappeared at 10 μM Fe^{2+}; this discrepancy is due to the rough concentrations used in preliminary tests and subsequent overestimation of enzyme concentration or underestimation of metal concentration. Interpretation of the data is described in Section 3.

To prevent the Fe^{2+} from being spontaneously oxidized to Fe^{3+} in significant amounts, only fresh solutions were used. Solid $FeCl_2$ was dissolved and mixed immediately with the apo-enzyme. Measurements were done within 30–60 minutes after the mixing to ensure that the equilibrium state would be obtained and that no significant oxidation of Fe^{2+} would have time to occur. It was known that the oxidation is slow at pH less than 7[3] and that the presence of various anions at millimolar concentrations slows it down further by up to an order of magnitude[4], so the oxidation would be negligible within an hour.

Table S1. Parameters of sample injection, acquisition and low vacuum stage used in mass spectrum measurements with the ESI FT-ICR MS (Bruker Solarix XR).

**Parameter**	**Value (denatured state)**	**Value (native state)**
Sample flow rate	120 μl/h	120 μl/h
Data size	2097152 (2M)	2097152 (2M)
Mass range	m/z 589.7–3000.0	m/z 202.7–6000.0
Averaged scans	150	100–200 (depending on sample)
Ion accumulation time	0.800 s	1.700 s
Capillary voltage	4500 V	4500 V
End plate offset voltage	−500 V	−500 V
Drying gas temperature	200.0 °C	80.0 °C
Drying gas flow rate	4.0 l/min	4.0 l/min
Nebulizer pressure	1.0 bar	1.0 bar
Ion polarity	Positive	Positive
Capillary exit voltage	220.0 V	270.0 V
Capillary exit voltage (high)	200.0 V	200.0 V
Deflector plate voltage	200.0 V	250.0 V
Funnel 1 voltage	150.0 V	200.0 V
Funnel 2 voltage	6.0 V	6.0 V
Skimmer 1 voltage	80.0 V	150.0 V
Skimmer 2 voltage	5.0 V	5.0 V
DC bias	0.0 V	0.0 V
Funnel RF amplitude	150.0 Vpp	150.0 Vpp
Octopole frequency	5 MHz	5 MHz
Octopole RF amplitude	350.0 Vpp	350.0 Vpp
Collision voltage (entrance)	−1.5 V	1.8 V
Collision RF frequency	2 MHz	2 MHz
Collision RF amplitude	1600.0 Vpp	1500.0 Vpp
DC extract bias (entrance)	−0.2 V	0.5 V
Time of flight	1.000 ms	1.500 ms
Transfer line frequency	4 MHz	2 MHz
Transfer line RF amplitude	350.0 Vpp	300.0 Vpp

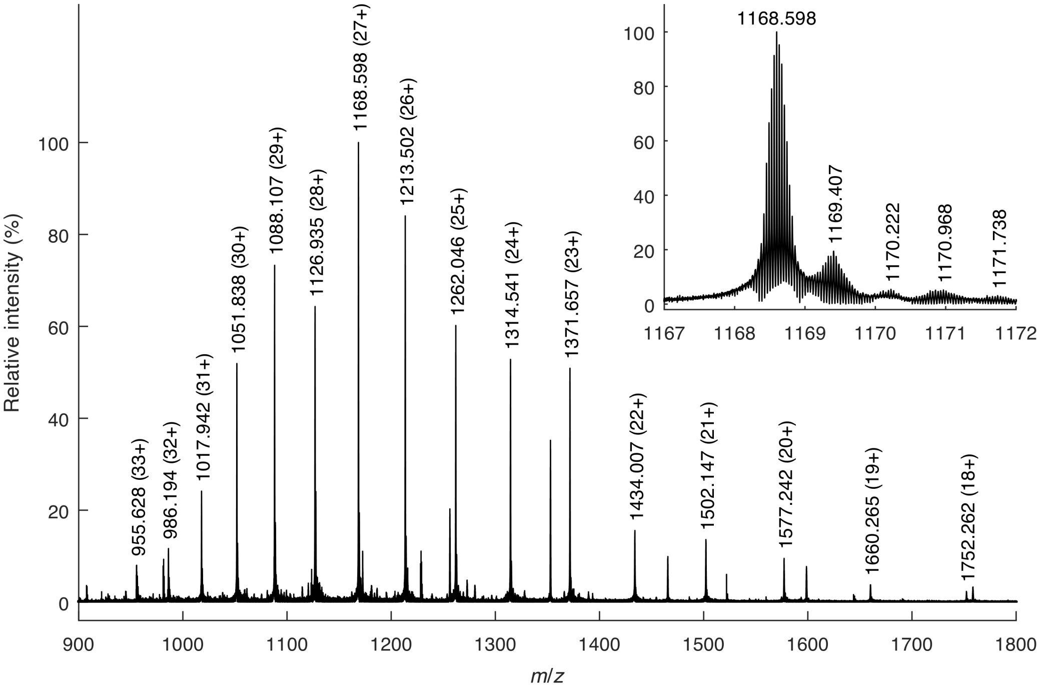

Figure S1. Mass spectrum of *Cc* XylC in denatured state. The inset is a magnification of the strongest 27+ charge state.

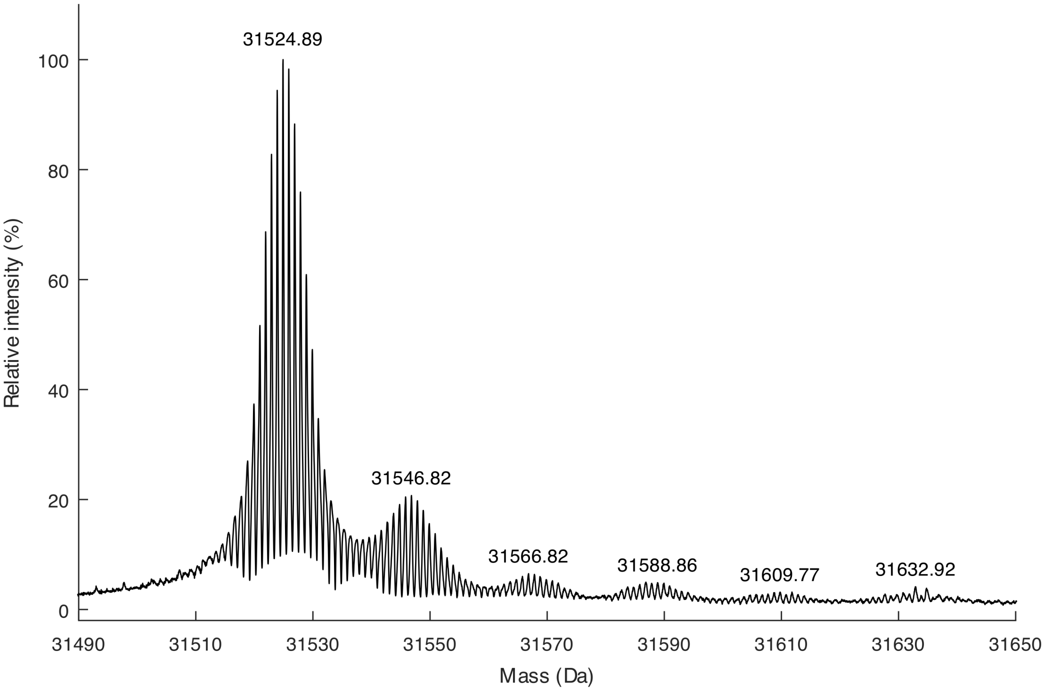

Figure S2. Charge state deconvolution of the denatured mass spectrum in Figure S1, showing the neutral mass of the enzyme.

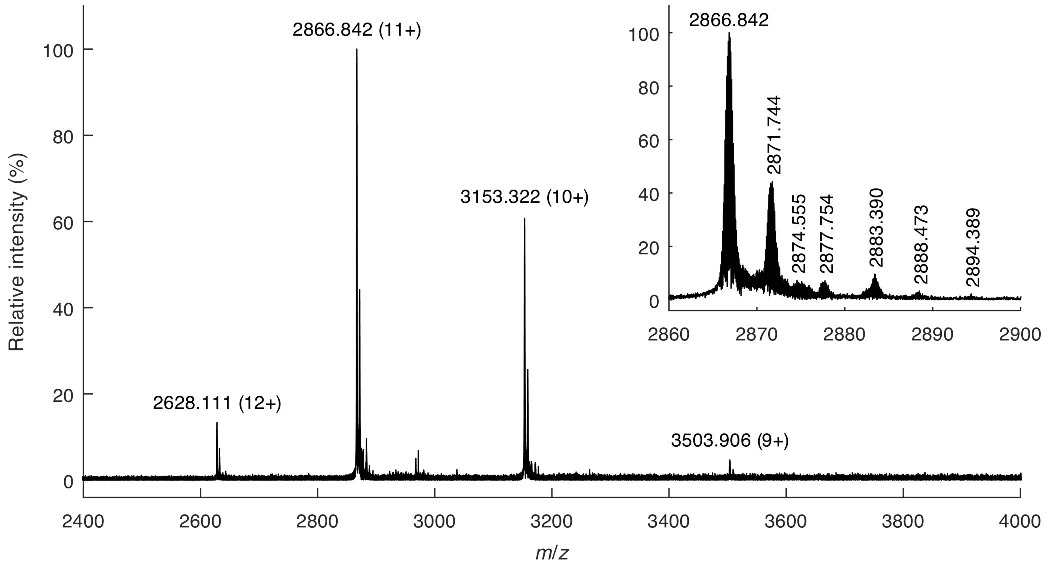

Figure S3. Native mass spectrum of the untreated *Cc* XylC. The inset is a magnification of the strongest 11+ charge state.

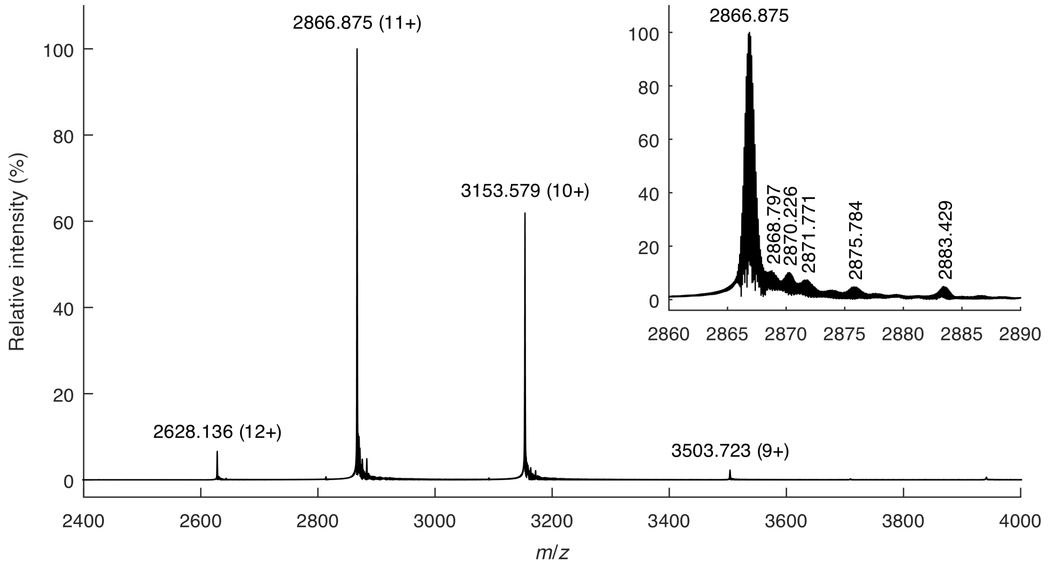

Figure S4. Native mass spectrum of *Cc* XylC in apo-form. The inset is a magnification of the strongest 11+ charge state.

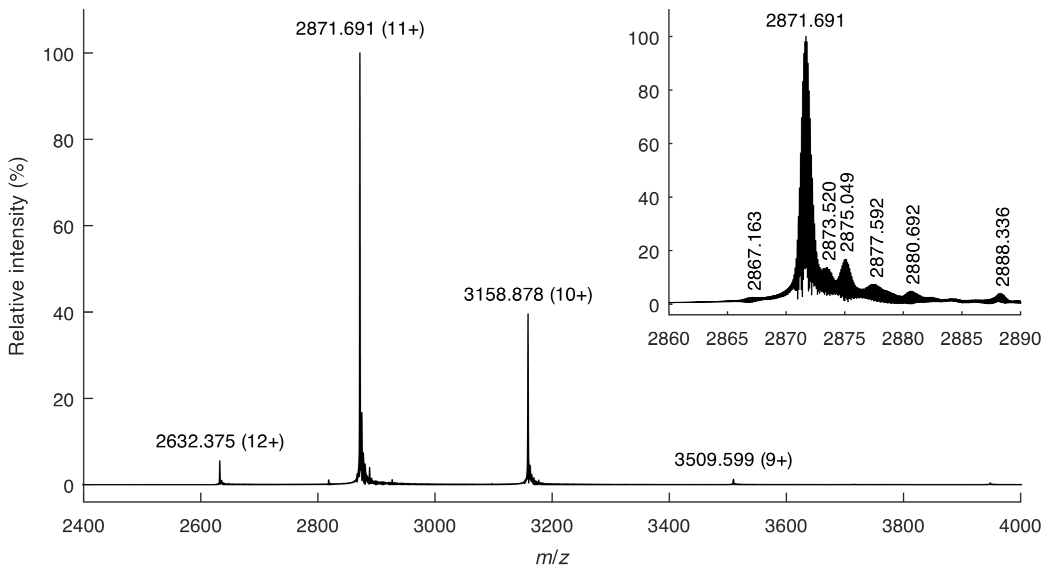

Figure S5. Native mass spectrum of *Cc* XylC in holo-form. The inset is a magnification of the strongest 11+ charge state.

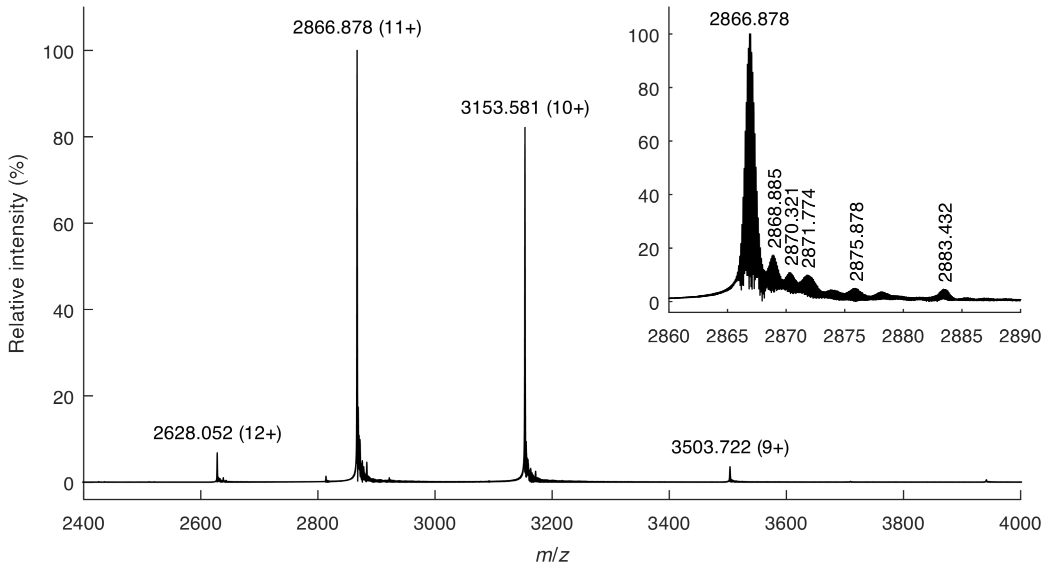

Figure S6. Native mass spectrum of *Cc* XylC with Zn^{2+} present, representative of the results when metal ions were added, excluding Fe^{2+} and Cu^{2+}: the enzyme is all in apo-form. The inset is a magnification of the strongest 11+ charge state.

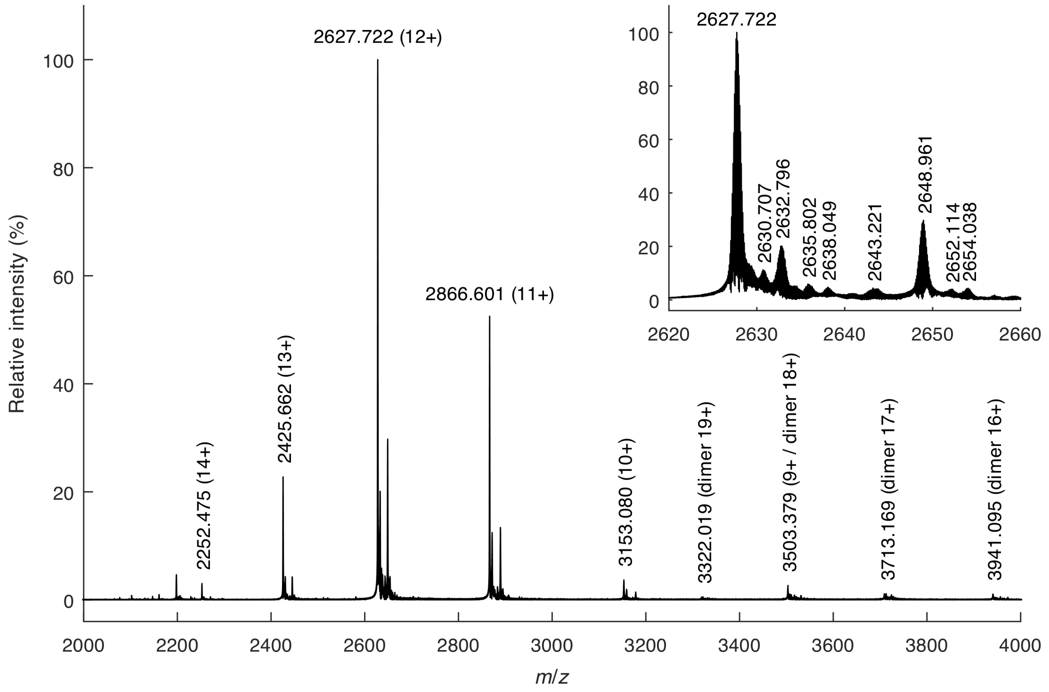

Figure S7. Native mass spectrum of *Cc* XylC with Cu^{2+} present, showing that copper ions bind to the enzyme nonspecifically. The inset is a magnification of the strongest 12+ charge state.

2 ESI QIT MS experimental procedures

The instrument used for reaction progress monitoring was a Bruker Esquire 3000plus quadrupole ion trap (QIT) mass spectrometer (Bruker Daltonik GmbH, Bremen, Germany), also equipped with an ESI source. The ion source pressure was ∼3 mbar, and the cell pressure was $\sim 1 \cdot 10^{-5}$ mbar. Measurements were done in chromatogram mode: the instrument took scans continuously (about once per second). Important measurement parameters are shown in Table S2.

Samples were prepared in 10 mM ammonium acetate solution. Each sample contained 0.25 mM lactone – either D-glucono-1,5-lactone (99.5 %, Merck) or D-xylono-1,4-lactone (95 %, Sigma-Aldrich) – and 1.0 mM xylitol which was added as an internal standard. The concentrations were chosen so that the intensities of the signals of the product (gluconic or xylonic acid) and xylitol were roughly equal. Due to rapid nonenzymatic hydrolysis, the sample measurements were started immediately after dissolving the lactone to the sample solution. For the reactions with iron present, 10 µM $FeCl_2$ was added, and for the enzymatic reactions, 0.5 µM EDTA-treated apo-enzyme was also added.

Table S2. Parameters of sample injection, acquisition and low vacuum stage used in reaction progress measurements with the ESI QIT MS (Bruker Esquire 3000plus).

**Parameter**	**Value**
Sample flow rate	100 µl/h
Mass range	m/z 30–400
Averaged scans	10
Maximum ion accumulation time	0.200 s
Drying gas temperature	150 °C
Drying gas flow rate	3.00 l/min
Nebulizer pressure	6.00 psi (0.414 bar)
Ion polarity	Negative
Capillary voltage	4000 V
Capillary exit voltage	−60.0 V
Capillary exit block	200.0 V
Skimmer voltage	−40.0 V
Skimmer block	−40.0 V
End plate offset voltage	−500 V
Octopole 1 voltage	−12.00 V
Octopole 2 voltage	−1.70 V
Octopole RF amplitude	103.0 Vpp

3 Determination of iron affinity to *Cc* XylC

The reaction equation of the specific binding of a metal ion M (here Fe^{2+}) to the active center of an enzyme is

$$\text{apo-enzyme} + \text{M} \rightleftarrows \text{holo-enzyme}. \tag{1}$$

The mass spectra of the protein samples were analyzed using DataAnalysis 5.0 software by Bruker. In order to determine the iron affinity to the *Cc* XylC, the fractional protein saturation B_C (the proportional amount of the holo-enzyme out of all enzyme) for each sample was calculated using the signal intensities of the apo- and holo-enzymes obtained from the mass spectra. The water-bound forms of the holo-enzyme were taken into account as well. Summing the intensities up over the observed charge states, this resulted in the total intensities of the apo- and holo-enzymes (ΣI_apo and ΣI_holo) for each sample. The fractional saturation is then

$$B_\mathrm{C} \equiv \frac{[\text{holo-enzyme}]}{c_\mathrm{enzyme}} = \frac{\Sigma I_\mathrm{holo}}{\Sigma I_\mathrm{apo} + \Sigma I_\mathrm{holo}}. \tag{2}$$

The intensities of the signals are assumed to be proportional to the concentrations of the corresponding species. The intensities were obtained using the SNAP peak finding algorithm which evens out noise in the isotopic distributions and gives good estimates of the monoisotopic masses and the intensities of protein signals. This is especially useful when signals are weak and signal-to-noise ratios are low.

For each sample, total enzyme concentration c_enzyme, total iron concentration c_M and fractional saturation B_C are known. Free iron concentration [M] is calculated using the formula

$$[\mathrm{M}] = c_\mathrm{M} - B_\mathrm{C} \cdot c_\mathrm{enzyme}. \tag{3}$$

The variables [M] and B_C are correlated by the 1:1 binding model

$$B_\mathrm{C} = \frac{B_\mathrm{max} \cdot [\mathrm{M}]}{K_\mathrm{d} + [\mathrm{M}]} \tag{4}$$

where K_d is the dissociation constant and B_max is the level of maximum saturation. This function was fitted to the data points using unweighted orthogonal distance regression in Origin Pro 2018 software[5]. The algorithm minimises the sum of squared orthogonal distances between the curve and the data points and optionally allows giving weights to both coordinates of each data point. Weighting the data points using estimated uncertainties was attempted but did not have a significant effect on the result. The program gave the confidence intervals of the fitted parameters with a 95 % level of confidence:

$$K_\mathrm{d} = (5.0 \pm 1.3) \cdot 10^{-7}\ \mathrm{mol/l},$$
$$B_\mathrm{max} = 0.966 \pm 0.009.$$

The data points and the fitted curve are shown in Figure 2 in the article.

4 Monitoring the nonenzymatic and enzymatic lactone hydrolysis

The ion chromatograms of the lactone hydrolysis reactions were analyzed in DataAnalysis 3.3 software by Bruker and exported as line spectra. The signal intensities of the product acid and xylitol were extracted using a custom script and opened in GNU Octave for calculations. These discrete intensity curves were trimmed so that unreliable values in the beginning and in the end were eliminated, and the time of starting the respective reaction was subtracted from each time point, resulting in intensities I_{acid} and I_{xylitol} over time t. To eliminate the majority of noise in the signal intensities, the numbers of data points were reduced by averaging the raw data points over time intervals of constant length, and this length for each respective reaction was chosen to be roughly one fifth of the estimated reaction half-life.

An acid "concentration" value c' is defined as

$$c'(t) = \frac{I_{\text{acid}}(t)}{I_{\text{xylitol}}(t)}. \tag{5}$$

Assuming that signal intensities are proportional to concentrations and that xylitol concentration is constant, c' is proportional to the concentration of the acid.

The rate equation of the pseudo-first-order reaction

$$\text{lactone (L)} + \text{water} \longrightarrow \text{acid (A)} \tag{6}$$

is

$$-\frac{\mathrm{d}c_{\text{L}}(t)}{\mathrm{d}t} = \frac{\mathrm{d}c_{\text{A}}(t)}{\mathrm{d}t} = kc_{\text{L}}(t) = k\left[c_{\text{L}}(0) - c_{\text{A}}(t)\right] \tag{7}$$

where k is the reaction rate constant, and c_{L} and c_{A} are true concentrations of lactone and acid respectively. Solving the differential equation of the second and the fourth expression leads to the integrated rate equation

$$c_{\text{A}}(t) = c_{\text{L}}(0) \cdot (1 - e^{-kt}). \tag{8}$$

During the reaction, concentration c_{A} will start from zero and rise up to the maximum level $c_{\text{L}}(0)$ which is the initial concentration of the lactone. This equation can also be written in terms of c':

$$c'(t) = c'(\infty) \cdot (1 - e^{-kt}). \tag{9}$$

This function can be fitted to data points (t, c'), and the fit will yield the rate constant k as well as the maximum level $c'(\infty)$.

The fit was done using GNU Octave with loaded Octave Forge packages `io`, `optim`, `statistics` and `struct` which are required for using the `nlinfit` function. Given values of t and c' in column vectors `at` and `ac` respectively, the following commands fit the function `curve`

from Equation (9) to the data points and return the values and uncertainties of k (`k` and `dk`) and $c'(\infty)$ (`cinf` and `dcinf`).

```
curve = @(b, t) b(2) * (1 - exp(-b(1) * t));
[beta R J covb] = nlinfit(at, ac, curve, [0.1 1]);
delta = sqrt(diag(covb)) * tinv(0.5 + 0.95 / 2, length(ac) - 2);
k = beta(1), dk = delta(1), cinf = beta(2), dcinf = delta(2)
```

The function `nlinfit` returns, as well as the parameters in vector `beta`, the variance-covariance matrix `covb` which is used to estimate the standard deviations of the parameters. They are multiplied by the critical value, given by the function `tinv`, where the two-tailed integral of Student's t-distribution with the appropriate degrees of freedom reaches 0.95, to get uncertainties with a 95 % level of confidence. The ion chromatograms and the fitted curves are shown in Figure S8.

Rate constants are not very illustrative, and so half-lives of lactone were also calculated for an alternative comparison of reaction progresses. Assigning $c_{\mathrm{A}}(t) = 0.5 \cdot c_{\mathrm{L}}(0)$ to Equation (8) and solving for t results in the half-life $t_{1/2}$:

$$t_{1/2} = \frac{\ln 2}{k} \approx \frac{0.6931}{k}. \tag{10}$$

In multiplication and division, uncertainty Δ propagates so that relative uncertainty remains:

$$\frac{\Delta t_{1/2}}{t_{1/2}} = \frac{\Delta k}{k}, \tag{11}$$

which results in

$$\Delta t_{1/2} = \frac{\ln 2 \cdot \Delta k}{k^2} \approx \frac{0.6931 \cdot \Delta k}{k^2}. \tag{12}$$

The *Cc* XylC catalyzed lactone hydrolysis is assumed to follow the Michaelis–Menten model[6,7]

$$-\frac{\mathrm{d}c_{\mathrm{L}}(t)}{\mathrm{d}t} = \frac{\mathrm{d}c_{\mathrm{A}}(t)}{\mathrm{d}t} = \frac{k_{\mathrm{cat}} \cdot c_{\mathrm{E}} \cdot c_{\mathrm{L}}(t)}{K_{\mathrm{M}} + c_{\mathrm{L}}(t)} \tag{13}$$

where k_{cat} is the turnover number, K_{M} is the Michaelis constant and c_{E} is the enzyme concentration. When $c_{\mathrm{L}} \ll K_{\mathrm{M}}$, the denominator approximately equals K_{M} and the equation becomes similar to the pseudo-first-order rate equation (7). This can be assumed to be true in this case since the integrated first-order rate equation appears to fit well to the data. Therefore, an estimate of the ratio $k_{\mathrm{cat}}/K_{\mathrm{M}}$ (specificity constant) can be calculated when the rate constant k and c_{E} are known:

$$\frac{k_{\mathrm{cat}}}{K_{\mathrm{M}}} = \frac{k}{c_{\mathrm{E}}}. \tag{14}$$

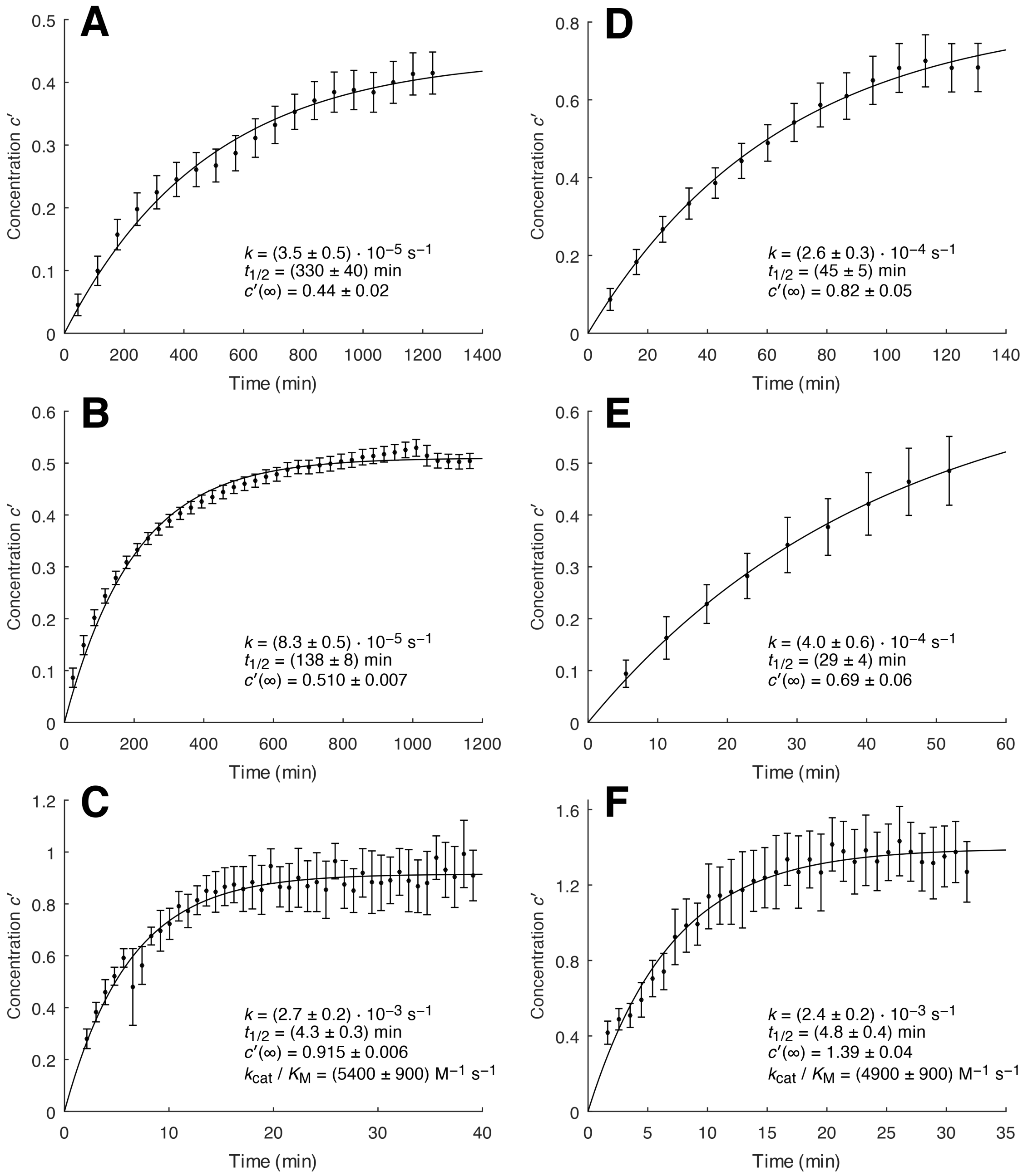

Figure S8. Measured reaction progresses (concentrations c', proportional to true concentrations, as defined in Equation (5)) and fitted progress curves (Equation (9)) for a) 0.25 mM D-xylono-1,4-lactone, b) 0.25 mM D-xylono-1,4-lactone, 10 µM Fe^{2+}, c) 0.25 mM D-xylono-1,4-lactone, 10 µM Fe^{2+}, 0.5 µM *Cc* XylC, d) 0.25 mM D-glucono-1,5-lactone, e) 0.25 mM D-glucono-1,5-lactone, 10 µM Fe^{2+}, f) 0.25 mM D-glucono-1,5-lactone, 10 µM Fe^{2+}, 0.5 µM *Cc* XylC. Error bars represent the standard deviations of the sets of raw data points from which the data points were averaged.

The uncertainties of k and c_E cannot be assumed to be uncorrelated, and their covariance cannot be determined. Therefore, the uncertainty of the ratio is estimated using the formula for maximum total uncertainty:

$$\Delta f(x_1, x_2, \ldots, x_N) \leq \sum_{i=1}^{N} \left| \frac{\partial f}{\partial x_i} \right| \Delta x_i, \tag{15}$$

which results in

$$\Delta \left(\frac{k_\mathrm{cat}}{K_\mathrm{M}} \right) = \frac{\Delta k}{c_\mathrm{E}} + \frac{k \cdot \Delta c_\mathrm{E}}{c_\mathrm{E}^2}. \tag{16}$$

The uncertainty Δc_E is estimated to be $0.1 \cdot c_\mathrm{E}$ (10 % relative uncertainty) with a 95 % level of confidence, so this becomes

$$\Delta \left(\frac{k_\mathrm{cat}}{K_\mathrm{M}} \right) = \frac{\Delta k + 0.1 \cdot k}{c_\mathrm{E}}. \tag{17}$$

5 References

(1) Boer, H.; Andberg, M.; Pylkkänen, R.; Maaheimo, H.; Koivula, A. In vitro reconstitution and characterisation of the oxidative D-xylose pathway for production of organic acids and alcohols. *AMB Express* **2019**, *9*, 48.

(2) Eaton, J. W.; Bateman, D.; Hauberg, S.; Wehbring, R. GNU Octave version 5.2.0 manual: a high-level interactive language for numerical computations. `https://www.gnu.org/software/octave/doc/v5.2.0/` (accessed Aug 27, 2020).

(3) Morgan, B.; Lahav, O. The effect of pH on the kinetics of spontaneous Fe(II) oxidation by O_2 in aqueous solution – basic principles and a simple heuristic description. *Chemosphere* **2007**, *68*, 2080–2084.

(4) Millero, F. J. The effect of ionic interactions on the oxidation of metals in natural waters. *Geochim. Cosmochim. Acta* **1985**, *49*, 547–553.

(5) *Origin Pro*, version 2018; OriginLab Corporation: Northampton, MA, 2018.

(6) Marangoni, A. G. Characterization of enzyme activity. *Enzyme kinetics: A modern approach*, 1st ed.; John Wiley & Sons: Hoboken, NJ, 2003; pp 44–60.

(7) Michaelis, L.; Menten, M. L. Die Kinetik der Invertinwirkung, *Biochem. Z.* **1913**, *49*, 333–369.
